# Shift in indications for radiotherapy during the COVID-19 pandemic? A review of organ-specific cancer management recommendations from multidisciplinary and surgical expert groups

**DOI:** 10.1186/s13014-020-01579-3

**Published:** 2020-06-03

**Authors:** Dirk Vordermark

**Affiliations:** Department of Radiation Oncology, Martin Luther University Halle-Wittenberg, Ernst-Grube-Str. 30, 06120 Halle / Saale, Germany

**Keywords:** COVID-19, Radiotherapy, Pandemic, Surgery

## Abstract

To examine, if a shift in indications for radiotherapy is to be expected in the context of the COVID-19 pandemic, the database Pubmed was searched for multidisciplinary or surgical expert recommendations on the role of radiotherapy in modified treatment concepts. Increased use of radiotherapy or chemoradiation instead of surgical treatment was recommended for defined patient groups with head-and-neck cancer, lung cancer, cervix cancer, esophageal cancer and prostate cancer. Omission of radiotherapy was considered in elderly patients with low-risk breast cancer and in early-stage Hodgkin’s lymphoma. Only adjustments to the timing of radiotherapy were discussed for sarcoma and rectal cancer. Emerging recommendations on multidisciplinary cancer treatment concepts during the COVID-19 pandemic indicate a shift in radiotherapy indications and a potentially increased demand for radiotherapy.

## Introduction

Responding to the ongoing COVID-19 pandemic, recommendations regarding the use of radiotherapy for cancer treatment and the reduction of risks in the radiotherapy process under the impact of limited resources have been presented from the radiation oncology community, including a recent statement from German-speaking university radiation oncology departments [1].

Radiotherapy expert groups have published specific recommendations on radiotherapy use in response to COVID-19 in selected tumor entities including breast cancer, head-and-neck cancer, prostate cancer and lung cancer [2–6], often focusing on the acceptability of hypofractionated concepts to spare resources. In addition, the impact of the COVID outbreak on the delivery of radiotherapy has been reported from most severely affected countries including China and Italy [7, 8] and a rapid guideline on radiotherapy delivery has been published in the United Kingdom [9].

In addition, multidisciplinary expert groups around the globe have published statements on the use of specific treatment modalities and their combinations during the pandemic, in particular addressing situations of limited availability of cancer surgery and related postoperative intensive care as well as reduced capacity of inpatient supportive care as part of intensive medical cancer therapy.

This work focusses on published site-specific cancer treatment recommendations from multidisciplinary and surgical expert groups and the question if radiotherapy will be required more or less frequently under the conditions of the pandemic.

## Methods

The database Pubmed was searched with the strategy [(COVID or coronavirus) and radiotherapy] and all publications listed in Pubmed by April 27, 2020, were reviewed. Only publications giving recommendations on the role of radiotherapy in the overall treatment concept (as elements of combined-modality concepts or as an alternative to other, e. g. surgical approaches) were reviewed. Papers from national or international groups of radiation oncology experts, concentrating on the choice of specific radiotherapy regimens under varying conditions of the COVID-19 pandemic, were excluded.

## Results

The Pubmed search strategy identified 40 publications which were reviewed. Recommendations from multidisciplinary or surgical expert groups on the role of radiotherapy in treatment concepts during the COVID-19 pandemic were included. The remaining publications were general or cancer-type specific recommendations by radiotherapy expert groups or reports of individual experiences in the management of cancer patients with COVID-19.

### Head and neck cancer

A thorough review of treatment concepts for head-and-neck cancer in the light of risks posed by the COVID-19 pandemic was presented by an interdisciplinary expert group from the University of Texas Southwestern [10]. Surgical treatment was seen as related to a particularly high number of risks including the combination of aerosol-generating events (e. g. coughing) and aerosol-generating procedures in the operating room in patients for whom the status of COVID-19 infection may be unclear (including asymptomatic, untested patients and patients with false negative tests). While making specific recommendations on the use of personal protection equipment in defined surgical risk situations, the authors “temporarily” favor radiation with or without chemotherapy wherever oncologic outcomes are equivalent to surgery plus adjuvant therapy.

### Gastrointestinal cancer

A surgical expert group from Rouen, France, recommended strategies for oncological surgery of gastrointestinal cancer during the pandemic [11]. As a general rule, they suggested to prefer non-surgical treatment for“ non-urgent” cases. Regarding rectal cancer, omitting surgery was not considered, but postponing its timepoint e. g. by extending the time interval between the end of long-course chemoradiation and surgery up to 12 weeks was suggested. For esophagogastric cancers treated with neoadjuvant chemotherapy or chemoradiation, deferral of gastrectomy or esophagectomy was considered, e. g. by prolonging the neoadjuvant phase by maintenance chemotherapy. Switching to radical chemoradiation without surgery (for esophageal cancer) was not recommended by this group.

Another treatment recommendation from a UK group composed predominantly of radiation oncologists focused on the general management of esophageal cancer under the conditions of increased intensive-care unit (ICU) bed occupancy by COVID-19 and limited capacity for surgical intervention [12]. They suggested to consider definitive chemoradiotherapy, rather than neoadjuvant chemoradiation followed by surgery, as the most appropriate curative option for both esophageal adenocarcinoma and squamous cell carcinoma.

### Gynaecologic cancer

The French FRANCOGYN group of the National College of French Gynecologists and Obstetricians (CNGOF) published recommendations on the management of gynecologic cancer during the pandemic [13]. They recommended for cervix cancer, that radiotherapy and concomitant radiochemotherapy should replace surgery as first-line treatment whenever possible. For endometrial and vulvar cancer, the indication for surgery is confirmed even under COVID-19 conditions, with the option to delay hysterectomy in low-risk endometrial cancer.

### Sarcoma

The French Sarcoma Group recommended to adhere, in principle, to the European Society of Medical Oncology (ESMO) Clinical Practice Recommendations in patients without COVID-19 symptoms [14]. In particular, they suggested not to delay surgery in patients with operable grade 2–3 soft tissue sarcoma. For cases of high-risk surgery such as in retroperitoneal sarcoma where postoperative ICU capacities are questionable, preoperative radiotherapy or chemotherapy was considered. It was recommended not to delay adjuvant radiation therapy for soft tissue sarcoma.

### Lung cancer

While no paper was identified using the above search strategy, a recommendation from the Lung Cancer Center of the Oncology Institute of Southern Switzerland was retrieved by an exploratory search [15]. Here, the risk of lung cancer progression (high for stage T4 any N and for N2 any T as well as for oligometastases) and the risk of COVID-19 infection (high if older than 70 years in combination with > 2 associated diseases or with immunosuppression) were classified and it was recommended to consider exclusive nonsurgical treatment in patients with both high risk of cancer progression and of COVID-19 infection. Essentially, this would include elderly comorbid patients with locoregionally advanced tumors or oligometastatic disease.

### Prostate cancer

No papers fulfilling the stated criteria were found. However, an exploratory search identified one publication from Italian high-volume uro-oncologic centers looking at the level of priority and of perioperative risk of patients treated there to determine which patients could have surgery safely postponed or switched to non-surgical treatment under conditions of reduced availability of health care resources [16]. For instance, 33.9% of radical prostatectomies were classified as high-priority (defined essentially as prostatectomy in high-risk prostate cancer patients “not eligible for radiotherapy” and in locally advanced prostate cancer). The authors proposed that in all patients with less than high priority, uro-oncologic surgery could be postponed and even in the group of high-priority surgery, a “non-negligible proportion might be shifted to alternative treatment strategies”.

### Breast cancer

For the management of breast cancer, one recommendation from a panel of multidisciplinary experts was found by an exploratory search [17]. Apart from the consideration of moderately or extremely hypofractionated radiotherapy regimens, the group considered postponing postoperative radiotherapy of early breast cancer for up to 6 months (in low-risk patients) and to omit radiotherapy in elderly patients at low risk of recurrence due to the limited benefit of this group from radiotherapy and increased risk of a severe course of COVID-19 disease.

### Brain tumors

While no recommendations where found with the search strategy, one publication from an international and multidisciplinary expert group on the management of adult glioma patients was detected in an exploratory search [18]. Here, standard-of-care therapy was predominantly recommended for newly diagnosed IDH-wild-type and IDH-mutant glioma, with the option of considering shorter courses of radiotherapy and avoidance of temozolomide in IDH-wild-type MGMT unmethylated tumors.

### Lymphoma

A Brazilian task force commented on the management of lymphoid malignancies during the COVID-19 outbreak [19]. A recommendation regarding modifications of the use of radiotherapy was given for early-stage Hodgkin’s lymphoma. Here, the task force suggested that it could be considered to omit radiotherapy at the cost of 6–8% disease control.

## Discussion

A general approach toward assuring the availability of radiotherapy under conditions of limited access to ressources (including staff) during the COVID-19 pandemic in German-speaking radiation oncology departments and the potential use of shortened, i. e. hypofractionated radiotherapy protocols has recently been published in this journal [1]. In parallel, several national and international expert groups have developed rather specific recommendations on which modified radiotherapy protocols are acceptable under such conditions.

This work focuses on the broader aspect of multidisciplinary treatment concepts. For a number of defined entities, modified approaches with different roles of the individual treatment modalities have been recommended for use during the COVID-19 pandemic in a short period during March and April of 2020. While these statements typically represent the views of experts in the respective fields and cannot be generalized to all healthcare systems globally, three patterns emerge regarding a modified role of radiotherapy in multimodality treatment concepts.

In situations where radical radiotherapy or chemoradiation already achieved outcomes comparable to operative treatment (with or without adjuvant radiotherapy) under normal conditions, a preference for non-surgical treatment, and thus a potentially increased demand for radiotherapy, becomes apparent from the current review of multidisciplinary and surgical recommendations for cervix cancer, esophageal cancer, lung cancer, head-and-neck cancer and prostate cancer. At the same time, indications for radiotherapy with a limited improvement in oncologic outcomes are viewed critically under situations of limited health care resources by some expert groups, including radiotherapy of low-risk breast cancer in elderly patients and of early-stage Hodgkin’s lymphoma. In a third group of indications, the omission of radiotherapy or its potential to replace a surgical treatment has not been discussed in the multidisciplinary context related to the COVID-19 pandemic, but rather different modes of timing of radiotherapy and surgery have been proposed, this includes sarcoma and rectal cancer.

In parallel with the first submission of this manuscript, Nagar and Formenti concluded in their commentary that increasing use of radiotherapy for cancer management should be considered as this modality does not compete with resources needed for COVID-19 management (i. e. ICU capacities), can be delivered on an outpatient basis, often in few treatment sessions, and is less immunosuppressive than other treatment modalities [20].

The present review of publications was limited to statements published in peer-reviewed journals by April 27, 2020 and further potentially relevant papers may have been found with other search strategies. As the COVID-19 pandemic develops through distinct phases around the globe, the ability of individual health care systems to provide specific elements of multimodality cancer treatment may develop very dynamically. After a first peak of the pandemic in many European countries in the spring of 2020, it is difficult to predict to what extent standard-of-care cancer treatment can be assured in the near future.

It can be hoped that surgical and intensive-care capacities in many countries will permit oncologic surgery near pre-COVID-19 levels. However, in certain entities such as head-and-neck cancer treatment, the risks seen by surgical experts in operating on potentially COVID-19-positive patients may affect decision-making between surgery and radiotherapy even in the more distant future.

Radiation oncologists need to expect a potential shift in indications for radiotherapy as a result of the COVID-19 pandemic. This shift may in summary lead to an increased demand for radiotherapy.

## Data Availability

All references are cited in the manuscript, no additional data or material is involved.

## Abbreviations

CNGOF: National College of French Gynecologists and Obstetricians
ESMO: European Society of Medical Oncology (ESMO)
ICU: Intensive-care unit
